# Gene expression profiles alteration after infection of virus, bacteria, and parasite in the Olive flounder (***Paralichthys olivaceus*)**

**DOI:** 10.1038/s41598-018-36342-y

**Published:** 2018-12-24

**Authors:** Gyu-Hwi Nam, Anshuman Mishra, Jeong-An Gim, Hee-Eun Lee, Ara Jo, Dahye Yoon, Ahran Kim, Woo-Jin Kim, Kung Ahn, Do-Hyung Kim, Suhkmann Kim, Hee-Jae Cha, Yung Hyun Choi, Chan-Il Park, Heui-Soo Kim

**Affiliations:** 1 Department of Biological Sciences, College of Natural Sciences, Pusan National University, Busan, 46241 Republic of Korea; 2 Institute of Systems Biology, Pusan National University, Busan, 46241 Republic of Korea; 3 Center for Convergence Approaches in Drug Development (CCADD), Graduate School of Convergence Science and Technology, Seoul National University, Suwon, 16229 Republic of Korea; 4 Department of Chemistry, Center for Proteome Biophysics and Chemistry Institute for Functional Materials, Pusan National University, Busan, 46241 Republic of Korea; 5 Department of Aquatic Life Medicine, Pukyong National University, Busan, 48513 Republic of Korea; 6 Biotechnology Research Division, National Fisheries Research and Development Institute, 216 Gijanghaean-ro, Gijang-eup, Gijang-gun, Busan, 46083 Republic of Korea; 7 Theragen ETEX Bio Institute, Suwon, 16229 Republic of Korea; 8 Department of Parasitology and Genetics, Kosin University College of Medicine, Busan, 49267 Korea; 9 Department of Biochemistry, College of Oriental Medicine, Dongeui University, Busan, 47227 Korea; 10 Department of Marine Biology and Aquaculture, College of Marine Science, Gyeongsang National University, Tongyeong, 53064 Korea

## Abstract

Olive flounder (*Paralichthys olivaceus*) is one of economically valuable fish species in the East Asia. In comparison with its economic importance, available genomic information of the olive flounder is very limited. The mass mortality caused by variety of pathogens (virus, bacteria and parasites) is main problem in aquaculture industry, including in olive flounder culture. In this study, we carried out transcriptome analysis using the olive flounder gill tissues after infection of three types of pathogens (Virus; Viral hemorrhagic septicemia virus, Bacteria; *Streptococcus parauberis*, and Parasite; *Miamiensis avidus*), respectively. As a result, we identified total 12,415 differentially expressed genes (DEG) from viral infection, 1,754 from bacterial infection, and 795 from parasite infection, respectively. To investigate the effects of pathogenic infection on immune response, we analyzed Gene ontology (GO) enrichment analysis with DEGs and sorted immune-related GO terms per three pathogen groups. Especially, we verified various GO terms, and genes in these terms showed down-regulated expression pattern. In addition, we identified 67 common genes (10 up-regulated and 57 down-regulated) present in three pathogen infection groups. Our goals are to provide plenty of genomic knowledge about olive flounder transcripts for further research and report genes, which were changed in their expression after specific pathogen infection.

## Introduction

Olive flounder is one of main marine species having high economic value in the countries of East Asia. Because of industrial importance by increasing of demand, it is serious subject to understand the pathogenic infection and production performance of olive flounder. Mass mortality of fishes is the most severe problem, accompanying vast deficit in aquaculture farm. Efforts to prevent regrettable death have especially been conducted in immunology^1–3^. However, so far, it is hard to figure out cause of death exactly in short time because of variables of expansive marine ecosystem. Mass mortality of olive flounder was normally caused by diseases via various sources of infection such as virus, bacteria, or parasite from external environment.

Viruses depend on host cell ribosomes to produce their proteins, and sometime use host cell DNA and RNA polymerases for replication and transcription, respectively. Many viruses encode proteins that modify the host transcription or translation apparatus to favor the synthesis of viral proteins over those of the host cell. Among viruses, viral hemorrhagic septicemia virus (VHSV) is affiliated to *Novirhabdovirus* genus, which is a member of the *Rhabdoviridae* family^4^. The six gene were contained in the VHSV genome of about 11 K bases and each of them coded nucleoprotein (N), phosphoprotein (P), matrix protein (M), glycoprotein (G), nonstructural viral protein (NV), and RNA polymerase (L) in the following order 3’-N-P-M-G-NV-L-5’^4^. Infection of VHSV results in contagious viral hemorrhagic septicemia (VHS) in diverse fish species regardless of their inhabitation; seawater or freshwater^5^. In East Asia, a lot of infection cases into olive flounder have been reported steadily, since VHSV was detected in middle of 1990s^6–9^.

A variety of scuticociliates have been reported as cause of scuticociliatosis in marine species including turbot, guppy, and southern bluefin tuna^10–12^. In olive flounder, disease has been reported to be causing from various scuticociliates; *Uronema marinum, Pseudocohnilembus persalinus, Philasterides dicentrarchi, Miamiensis avidus*^13–16^. Interestingly, judging from infection experiments using various scuticociliates plus identification outcome of 8 isolates acquired from olive flounders with symptom of ulcers and haemorrhages, *Miamiensis avidus* was suggested as the major aetiologic agent of scuticociliatosis because of high pathogenicity and mortality rate compared with other scuticociliates^14,17^.

Infection of bacteria could sustain serious damage to fish. Streptococcosis is known to be caused by a variety of streptococcic species; *Streptococcus parauberis, Streptococcus iniae, Streptococcus difficilis, Lactococcus garvieae, Lactococcus piscium, Vagococcus salmoninarum, and Carnobacterium piscicola*, and has become major nuisance in olive flounder farms^18–21^. In particular, *Streptococcus iniae, Lactococcus garvieae, and Streptococcus parauberis* have been introduced to be related with Streptococcosis in olive flounder^19–23^.

The main issue of aquaculture industry is to reduce economic loss by preventing mortality of fish from various pathogens. A large number of immunologic studies have been proceeded about various immune-related gens against pathogen infection^3,24–27^. A huge quantity of genomic information from next generation sequencing (NGS) technique has been gradually increasing for the last few years, indicating that researchers could approach more comprehensive understanding view about genome of organisms than when they research a single gene level. With development of wide-sized analysis methods, it is not difficult to figure out change of gene expression level after any chemical treatment or environmental change. Recently, studies to identify large-scale genes were conducted in the olive flounder genome for researches about vaccine, gonadal development, and sex determination^28–30^. In particular, characterizing of immune-related genes was reported in olive flounder spleen tissue^31^. A lot of studies reported earlier were focused on gene expression analysis of single pathogen and specifically defined the expression pattern of limited genes^32–36^. Further, infection by two or more pathogens were reported in the olive flounder genome^37,38^. In order to solve these problem, we need plentiful genomic information to respond rapidly to multiple infection of pathogens. However, researches, which were comprehensively analysed about change of gene expression pattern by different type of pathogens, have not been reported in the olive flounder genome, so far.

In this research, we identified differentially expressed genes (DEGs) by transcriptome analysis and conducted gene ontology (GO) analysis with genes identified. Then, we tried to find important genes which showed consistently meaningful expression change in the results of three infection experiments. As a result, we determined 10 up-regulated genes and 57 down- regulated genes in common after infection of three pathogens. We aimed to provide essential genome information which is related with pathogen infection and explore the various consequences related to differential infections and find out the common strategies against specific candidates involved in disease progression in natural habitat of aquaculture.

## Results

### Statistical summary of transcriptome analysis

To profile gene expression after infection of three pathogens (VHSV, *Streptococcus parauberis*, and *Miamiensis avidus*), transcriptome analysis was conducted using gill tissues of olive flounders, respectively. We prepared twelve olive flounders (three un-infected individuals as control, three virus-infected, three bacteria-infected, and three parasite-infected individuals) to raise confidence. To gain the sufficient number of transcripts, twelve independent RNA samples acquired from normal and pathogen-infected olive flounder gill tissues were employed for construction of cDNA library. Then, these cDNA libraries were sequenced using Illumina HiSeq2500, generating the numbers of approximately 78.4 million, 65.2 million, and 45.7 million raw reads from three control samples, 44.0 million, 56.9 million, and 62.0 million raw reads from bacteria-infected samples, 41.2 million, 62.4 million, and 40.0 million raw reads from virus-infected samples, 39.6 million, 41.6 million, and 53.1 million raw reads from parasite-infected samples, respectively (Table 1). After trimming of low-quality reads and adaptor sequences, the number of clean reads acquired from control samples were average 62.3 million reads from control samples, average 53.5 million reads from bacteria-infected samples, average 48.0 million reads from virus-infected samples, and average 44.3 million reads from parasite-infected samples, respectively. Then, we checked gene coverage whether the reads that we acquired are sufficient for quantitative gene expression analysis (Supplementary Fig. 1). The clean reads were assembled into 120,880 transcript sequences acquired from transcriptome analysis, we identified total 40,100 genes involving novel 19,560 genes from transcript sequences using InterProScan database and non-redundant protein database in the NCBI (Table 2).

**Table 1:** Statistical summary of reads acquired from transcriptome analysis.

Description	Samples											
	U-I 1	U-I 2	U-I 3	B-I 1	B-I 2	B-I 3	V-I 1	V-I 2	V-I 3	P-I 1	P-I 2	P-I 3
*Reads*												
Number of raw reads	78,425,340	65,251,756	45,720,414	44,022,532	56,928,000	62,034,784	41,202,926	62,465,936	40,015,984	39,636,294	41,676,740	53,148,278
Number of clean reads	77,685,792	64,293,520	45,046,422	43,370,168	56,218,774	61,171,560	40,540,550	64,212,934	39,393,956	39,128,496	41,195,252	52,580,022
Number of mapped reads	35,293,624	34,011,030	21,768,778	23,241,737	25,467,484	26,640,040	25,215,623	37,509,022	19,159,186	6,587,353	13,259,405	11,685,297
Number of uniquely mapped reads	34,624,745	33,266,973	21,287,829	22,815,501	24,897,617	26,076,243	24,651,733	36,604,718	18,740,455	6,293,804	12,980,394	11,405,891
Mapping rate of unique reads	44.6%	51.7%	47.3%	52.6%	44.3%	42.6%	60.8%	59.8%	47.6%	16.1%	31.5%	21.7%

U-I: uninfected, B-I: bacteria-infected, V-I: virus-infected, P-I: parasite-infected.

**Table 2:** The number of genes and transcripts identified from transcriptome analysis.

Description	Samples												Summary
	U-I 1	U-I 2	U-I 3	B-I 1	B-I 2	B-I 3	V-I 1	V-I 2	V-I 3	P-I 1	P-I 2	P-I 3	
*Gene*													
Known	1,707	1,720	1,504	1,641	1,617	1,669	1,670	1,751	1,664	1,348	1,588	1,243	**1,969**
Known (New Isoforms)	18,564	18,567	18,561	18,562	18,564	18,567	18,563	18,568	18,563	18,539	18,563	18,501	**18,571**
Novel	19,380	19,527	19,066	19,474	19,338	19,495	19,528	19,547	19,461	18,443	19,287	17,765	**19,560**
TOTAL	39,651	39,814	39,131	39,677	39,519	39,731	39,761	39,866	39,688	38,330	39,438	37,509	**40,100**
*Transcript*													
Known	25,572	25,078	24,574	24,900	25,079	24,966	24,479	24,618	24,189	23,061	24,516	22,945	**27,216**
Novel	93,402	93,533	93,017	93,505	93,345	93,513	93,532	93,527	93,416	92,260	93,297	91,281	**93,664**
TOTAL	118,974	118,611	117,591	118,405	118,424	118,479	118,011	118,145	117,605	115,321	117,813	114,226	**120,880**

U-I: uninfected, B-I: bacteria-infected, V-I: virus-infected, P-I: parasite-infected.

### Identification of DEGs after viral infection

**Figure 1:**
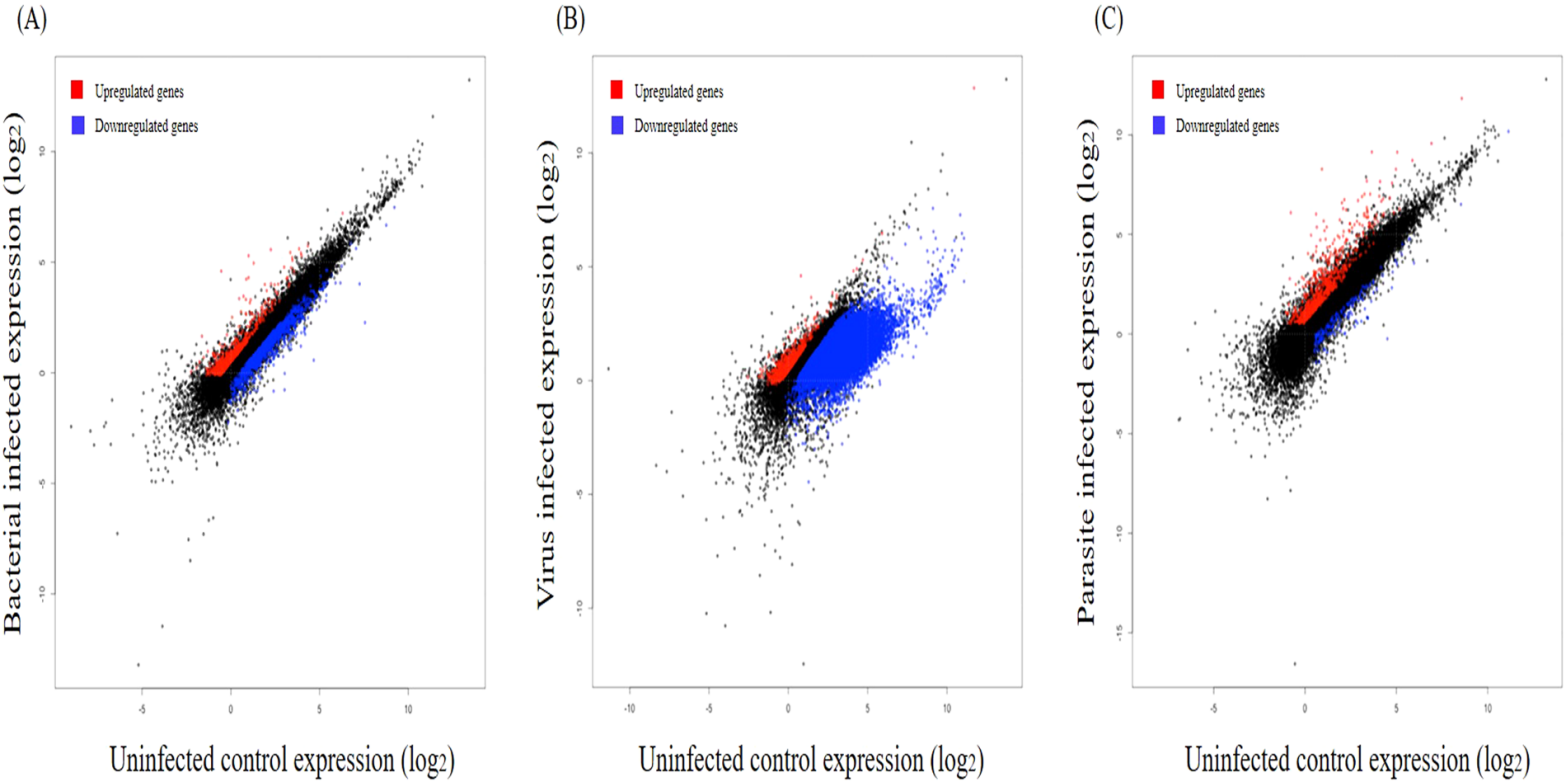
Global gene expression changes after infection by three pathogens. Genes expression in uninfected control samples plotted versus genes expression in bacterial infected samples (**A**), in virus infected samples (**B**), and in parasite infected samples (**C**). Genes changed after pathogen infection are colored in red and green for upregulated and downregulated, respectively (*p* < 0.05).

To figure out the effects of external pathogen for gene expression, we sorted out genes which showed expressional change after pathogens infection having *p*-value of <0.05 when compared with control sample. As shown in Table 3 and Fig. 1, the largest numbers of gene expression change were shown in VHSV infection group; total 12,415 DEGs were identified from transcriptome analysis. We showed information of DEGs derived from viral infection in Supplementary Table 1, as well as those after bacteria and parasite infection. In this result, we verified interesting phenomenon related in the number of DEGs after viral infection. Among 12,415 DEGs, total 11,051 DEGs were down-regulated after viral infection while 1,364 DEGs showed up-regulation due to viral infection, indicating the number of down-regulated DEGs strikingly outnumbered than those of up-regulated DEGs as 8.1 folds after viral infection in olive flounder genome. 9,699 of 12,415 DEGs were annotated by InterProScan database and non-redundant protein database in the NCBI and used for functional analysis in next step.

To explore the functional enrichment of these DEGs, we performed GO enrichment analysis using DAVID tool^39^ and sorted specially out the immune-related GO terms with *p-*value of <0.05. As a result, 134 DEGs were enriched in 9 GO terms, including ‘GO:0000045; autophagic vacuole assembly’ (*p* = 0.031353), ‘GO:0002376; immune system process’ (*p* = 0.024692), ‘GO:0006979; response to oxidative stress’ (*p* = 0.040376), ‘GO:0016032; viral process’ (*p* = 0.000325), ‘GO:0019221; cytokine-mediated signaling pathway’ (*p* = 0.016815), ‘GO:0030217; T cell differentiation’ (*p* = 0.032158), ‘GO:0033077; T cell differentiation in thymus’ (*p* = 0.024688), ‘GO:0042113; B cell activation’ (*p* = 0.037259), and ‘GO:0050853; B cell receptor signaling pathway’ (*p* = 0.020561), in GO category ‘biological process’ (Supplementary Table 2).

### Gene expression change pattern after bacterial infection

After analyzing DEGs and their functional prediction following viral infection, we identified the DEGs with *p*-value of <0.05 after infection with bacteria (*Streptococcus parauberis*) and parasite (*Miamiensis avidus*) (Table 3 and Fig. 1). First, infection of *Streptococcus parauberis* changed expression level of total 1,754 genes. Among them, 720 genes were upregulated, while 1,034 genes showed decreasing expression pattern. In case of bacterial infection, total number of DEGs after bacterial infection showed the number of one-seventh when compared with those of viral infection. Then, we carried out GO enrichment analysis with annotated 751 of 1,754 DEGs by genomic database and found that 11 DEGs were enriched in 5 immune-related GO terms {‘GO:0001865; NK T cell differentiation’ (*p* = 0.034926), ‘GO:0002302; CD8-positive, alpha-beta T cell differentiation involved in immune response’ (*p* = 0.033205), ‘GO:0032609; Interferon-gamma production’ (*p* = 0.000022), ‘GO:0032633; Interleukin-4 production’ (*p* = 0.034926), and ‘GO:0042060; Wound healing’ (*p* = 0.012764)} having *p-*value of <0.05 of ‘biological process’ GO category (Supplementary Table 2).

**Table 3:** The number of DEGs after pathogens infection.

Pathogen	Expression change to control		
	Up	Down	Total
Virus	1,364	11,051	12,415
Bacteria	720	1,034	1,754
Parasite	640	155	795

### Gene expression change pattern after parasite infection

Last, we checked how infection of *Miamiensis avidus* affected gene expression pattern in olive flounder genome and selected genes which showed expression change (Supplementary Table 2). We identified 795 DEGs caused by infection of *Miamiensis avidus*; 640 upregulated and 155 downregulated genes. By GO enrichment analysis with annotated 346 genes, we found only one immune-related GO term {GO:0050294; Steroid sulfotransferase activity, (*p* = 0.011417)} including one DEG.

### Distributional pattern of total DEGs acquired from transcriptome analysis

**Figure 2:**
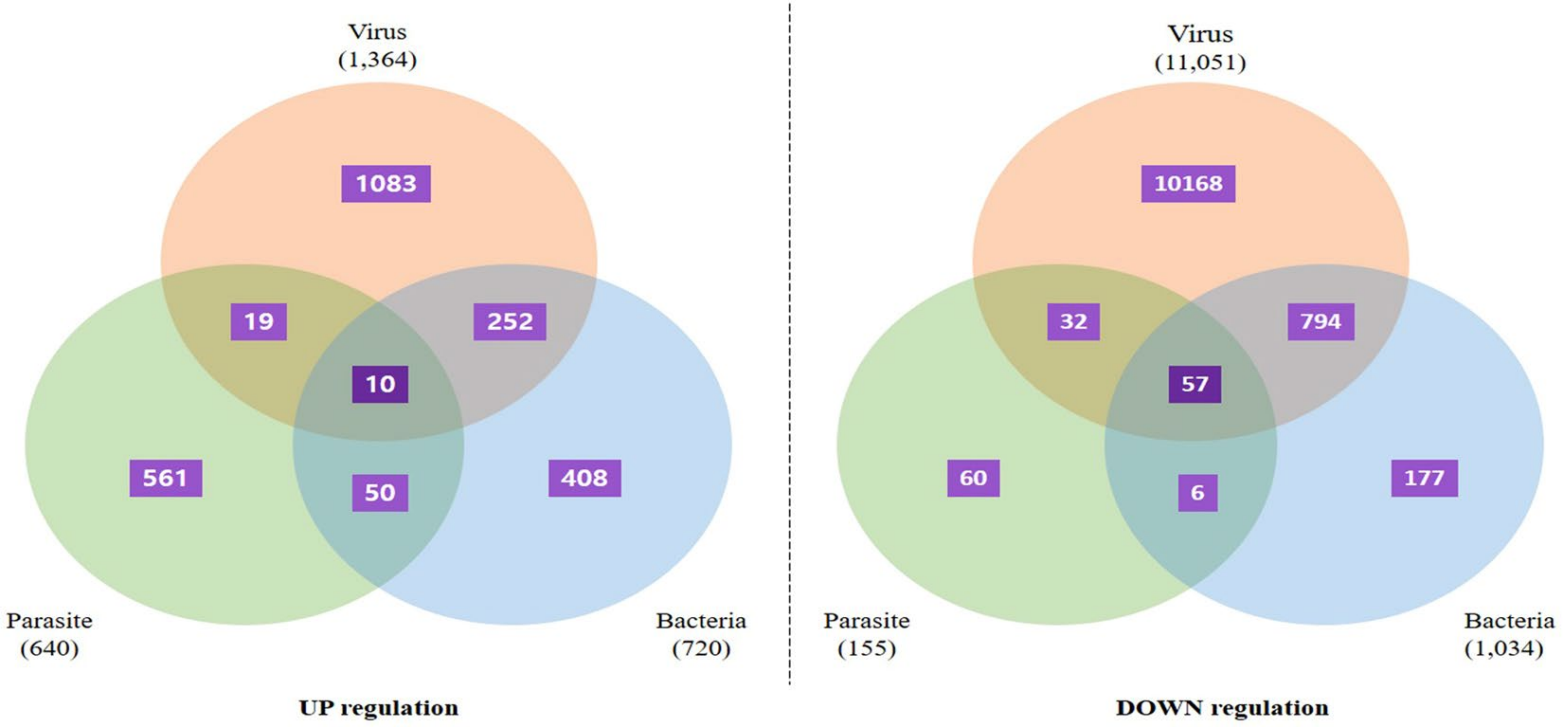
Distribution of DEGs in three pathogens group (bacteria, virus, and bacteria). Venn diagram show the number of DEGs among bacteria, virus, and parasite. 10 up-regulated and 57 down-regulated DEGs were overlapped in three pathogens group on common. The up-regulation of 1083 genes (virus), 408 genes (bacteria), and 561 genes (parasites). These genes were specific for each pathogen so can be used as candidate genes for vaccination or therapeutic agents. In case of the down-regulation of 10168 gene (virus), 177 gene (bacteria) and 60 gene (parasites) in infection of fish, these genes were specific for specific pathogens so can be used as a diagnosis marker for specific pathogens.

After comparison of gene expression level among twelve transcriptome analysis, we identified DEGs after pathogen infection. Then, we focused on selection of genes showing expression change pattern after three types of pathogen infection in common. As a result, we summarized 10 up-regulated genes and 57 down-regulated genes, respectively (Fig. 2). We analyzed these 67 DEGs to identify their gene symbol correctly using their sequences in non-redundant protein database of the NCBI database, and 37 DEGs were annotated (Table 4). We showed the rest of 30 unannotated DEGs in Supplementary Table 3.

**Table 4:** List of annotated DEGs among common gene after three pathogens infection.

Gene Expression Change (Control → Infection)	Gene Accession	Description	Fold change (log2) (Bacteria/control)	Fold change (log2) (Virus/control)	Fold change (log2) (Parasite/control)	Predicted gene symbol
UP	GENE19852.r1	Fibroblast growth factor 4 [Source:SWISS;Acc:P48804]	0.658	0.661	0.782	FGF4
Down	GENE00707.r1	Acidic mammalian chitinase [Source:SWISS;Acc:Q91XA9]	−0.540	−1.386	−1.367	Chia
	GENE16184.r1	Rab GDP dissociation inhibitor beta [Source:SWISS;Acc:P50395]	−1.739	−1.399	−1.218	GDI2
	GENE11810.r1	Neuronal acetylcholine receptor subunit alpha-7 [Source:SWISS;Acc:P22770]	−1.041	−1.566	−2.009	CHRNA7
	GENE04169.r1	Histidine decarboxylase [Source:SWISS;Acc:P23738]	−1.177	−1.173	−1.266	Hdc
	GENE11460	Semaphorin-3E [Source:SWISS;Acc:O42237]	−1.454	−2.466	−1.203	SEMA3E
	GENE21840.r1	Cysteine-rich secretory protein LCCL domain-containing 1 [Source:SWISS;Acc:Q8CGD2]	−0.705	−1.769	−1.146	Crispld1
	**GENE23270.r1**	**Cx9C motif-containing protein 4 [Source:SWISS;Acc:P56277]**	**−1.562**	**−3.848**	**−2.557**	CMC4*
	GENE06025.r1	Protein phosphatase 1 regulatory subunit 3C-B [Source:SWISS;Acc:Q6P950]	−0.829	−1.627	−1.308	ppp1r3cb
	**GENE18715.r1**	**Collagen alpha-2(I) chain [Source:SWISS;Acc:O46392]**	**−3.069**	**−5.000**	**−2.317**	COL1A2*
	GENE18863	2-aminoethanethiol dioxygenase [Source:SWISS;Acc:Q6PDY2]	−1.029	−2.165	−1.399	Ado
	GENE05041.r1	AP-1 complex subunit sigma-3 [Source:SWISS;Acc:Q7TN05]	−1.146	−1.544	−1.340	Ap1s3
	GENE03465	Homeobox protein Nkx-3.1 [Source:SWISS;Acc:Q99801]	−1.078	−3.351	−1.063	NKX3-1
	GENE24080.r1	Transmembrane protein 205 [Source:SWISS;Acc:A1L2F6]	−0.743	−2.695	−0.980	tmem205
	GENE14826.r1	Ethanolamine-phosphate phospho−lyase [Source:SWISS;Acc:Q7SY54]	−1.289	−1.961	−1.274	etnppl
	GENE01095.r1	Alcohol dehydrogenase 1 [Source:SWISS;Acc:P26325]	−0.750	−1.348	−0.906	ADH1_GADMC
	GENE12085.r1	Pyridine nucleotide-disulfide oxidoreductase domain−containing protein 2 [Source:SWISS;Acc:Q68FT3]	−0.953	−1.658	−0.839	Pyroxd2
	GENE22874.r1	Potassium voltage-gated channel subfamily KQT member 3 [Source:SWISS;Acc:Q8K3F6]	−0.616	−0.475	−0.785	Kcnq3
	GENE06563	Protein Hook homolog 3 [Source:SWISS;Acc:Q6GQ73]	−1.723	−4.109	−1.638	hook3
	GENE01318.r1	Mitochondrial peptide methionine sulfoxide reductase [Source:SWISS;Acc:P54149]	−0.693	−1.130	−0.953	MSRA
	GENE02237.r1	Anterior gradient protein 3 homolog [Source:SWISS;Acc:Q8TD06]	−1.288	−3.663	−1.523	AGR3
	GENE11050.r1	Viral T−cell receptor beta chain-like T17T-22 [Source:SWISS;Acc:P11364]	−1.211	−3.670	−1.053	V-TCR
	GENE09774	Claudin-12 [Source:SWISS;Acc:Q5R9K1]	−0.685	−2.185	−0.839	CLDN12
	GENE11823.r1	Proline-rich protein 5-like [Source:SWISS;Acc:A2AVJ5]	−0.924	−1.909	−0.949	Prr5l
	**GENE03568.r1**	**Urea transporter 2 [Source:SWISS;Acc:Q28614]**	**−2.107**	**−4.956**	**−2.405**	SLC14A2*
	GENE06547	Krueppel-like factor 5 [Source:SWISS;Acc:Q9Z0Z7]	−1.264	−2.379	−1.251	Klf5
	GENE09838.r1	Integrin alpha-10 [Source:SWISS;Acc:O75578]	−1.017	−2.154	−1.004	ITGA10
	GENE21626.r1	Hydroxycarboxylic acid receptor 2 [Source:SWISS;Acc:Q8TDS4]	−1.033	−2.299	−1.053	HCAR2
	GENE01511.r1	Ectonucleoside triphosphate diphosphohydrolase 5 [Source:SWISS;Acc:O75356]	−1.063	−1.592	−0.818	ENTPD5
	GENE15828.r1	Matrix metalloproteinase-20 [Source:SWISS;Acc:O18767]	−1.145	−2.128	−0.738	MMP20
	**GENE06887.r1**	**Osteocalcin [Source:SWISS;Acc:P02823]**	**−2.740**	**−3.546**	**−1.659**	bglap*
	GENE01596.r1	GTP cyclohydrolase 1 feedback regulatory protein [Source:SWISS;Acc:Q6PBT6]	−1.253	−2.255	−0.904	gchfr
	**GENE16851.r1**	**Aminopeptidase N {ECO:0000312|EMBL:ACZ95799.1} [Source:SWISS;Acc:O57579]**	**−1.504**	**−3.185**	**−2.428**	ANPEP*
	XLOC_002910	Trypsin-1 [Source:SWISS;Acc:P35031]	−1.401	−1.350	−2.147	TRY1_SALSA
	**XLOC_021180**	**Collagen alpha-1(I) chain [Source:SWISS;Acc:P02457]**	**−2.092**	**−5.204**	**−2.025**	COL1A1*
	XLOC_036089	AT-hook-containing transcription factor [Source:SWISS;Acc:Q80VW7]	−1.577	−2.290	−1.507	Akna
	XLOC_029355	Collagen alpha-3(VI) chain [Source:SWISS;Acc:P15989]	−1.301	−3.553	−0.819	COL6A3

*Six genes with asterisk are representive genes which have fold change (log2) of <−2.0 in at least two pathogen groups. *p* < 0.05.

## Discussion

With development of sequencing technique, numerous genomic researches have been reported to understand infection results by virus^28,31,40^, bactria^26,41,42^, and parasite^43^ in the olive flounder genome. A fundamental way to overcome disease outbreak from external pathogens is to approach from their genome level. It is essential to expand quantity of genomic information in pursuance of biological research about any target. Investigation of overall gene expression change after pathogen infection would provide clues of cause of biological damage. Gene regulation is essential for viruses, prokaryotes and eukaryotes as it increases the versatility and adaptability of an organism by allowing the cell to express protein when needed.

Phylogenetic diversity of pathogens (virus, bacteria and parasite) is also responsible for differential expression of genes in diseases. Each individual pathogen causes disease in a different way, which makes it challenging to understand the basic biology of infection. In this study, we understood the relation between three types of pathogen infection and differential gene expression in the olive flounder genome through transcriptome analysis, respectively. The diverse pathogens used in this study, carry specific antigenic variations, which refers to the specific mechanism by which an infectious agent infect the fish and progress the disease. Transcriptome analysis help us to understand the progression of disease in fish through pathogen infection based on diversity of pathogen (virus, bacteria and parasite). This study shows differentially expressed genes were up- and down-regulated at different extend in fish tissue. Interestingly, virus and bacteria have more down-regulated genes while parasite have more up-regulated genes. This data signifies the fact that fish immune system interacts with bacteria and virus with the same strategy, while with parasite different due to difference in mode of infections between them.

For efficient prevention against pathogen, it is important to understand which genes were activated/repressed after pathogen infection because their expression change means variation of metabolic system in body. In this study, we identified total number of 12,415 in VHSV infection group, 1,754 in *Streptococcus parauberis* infection group, and 795 DEGs from *Miamiensis avidus* infection group, respectively (Table 3). Given the difference in the number of DEGs among three pathogen groups, these results seemed that virus had the most impact on gene expression mechanism in the olive flounder genome among three pathogens. Interestingly, 11,051 DEGs (89% of all DEGs) showed down-regulated pattern after viral infection. This phenomenon that global gene expression was decreased by viral infection must cause pathogenic disease by affecting immune-related gene expression level, finally leads to death. This view was supported by our findings (Supplementary Table 2), which showed expression decrease pattern of all genes in the immune-related GO terms. Especially, GO terms in viral infection group showed that all genes tend to be down-regulated after pathogen infection, indicating loss of resistance against pathogens by down-regulating the expression of immune-related genes. Like this situation, functional information of genes acquired from GO enrichment could help researcher to figure out critical biological pathway against any external factor.

The immune mechanism in fishes is composed of a set of cellular and humoral system and divided into innate (inherit), and adaptive (acquired) substances. The understanding of fish immune system structure and function is essential for the development of new technologies and products to improve productivity. The transcriptome analysis bring exposure to basic difference in expression profile of all pathogens in the host. These differences were due to nature of parasite, their mode of infection, antigenic variations and many other factors. Additionally, along with all above differences, disease progressed in host due to external surface variation of pathogens (viral, parasite and bacterial) and their appropriate recognition by host immune systems for making the basis to initiate microbial clearance^44,45^. Disease research requires the knowledge of important key factors like method of avoiding host immune surveillance, antigenic variations, subversion of immune responses through phagocyte and inhibition of cytokines and chemokines in common with pathogen infections (viral, bacterial and parasite)^44,45^. On the other hand, the disease progression was different in accordance to type of pathogens. In case of viral infection, understanding of complement inhibition and blockade of cellular immunity is the most important, while parasite and bacterial infections required knowledge and research of innate pathway and acquired immunity^44,45^. Our analysis indicates the complexity and difference of expression profile could be due to all the above reasons. In addition, important basis of fish vaccine is depending on innate and adaptive immunity^46^. There were many vaccine types which depend upon antigens, live microorganisms or specific DNA segment of pathogens or polyvalent vaccines. All above vaccines required complete knowledge of pathogenicity and deep research of efficacy^47^. Our study clearly indicates about various immune and antigenic genes which can be chosen for pathways analysis and use for therapeutic agents or as some vaccine candidates (Supplementary Table 2).

Despite of their different infection pathway, we wondered common DEGs which were affected from infection. As shown in Fig. 2, total 67 DEGs (10 up-regulation and 57 down-regulation) were identified in common in three pathogens, and 1 of 10 up-regulated and 36 of 57 DEGs were annotated by genomic database (Table 4; Supplementary Table 3). These common genes can be used for diagnosis purpose as a candidate gene for any kind of pathogens (virus, bacteria and parasites). Here, we mentioned that representative six genes (ANPEP; Alanyl Aminopeptidase, Membrane, BGLAP; Bone gamma-carboxyglutamic acid-containing protein, CMC4; C-X9-C Motif Containing 4, COL1A1; Collagen Type I Alpha 1 Chain, COL1A2; Collagen Type I Alpha 2 Chain, SLC14A2; Solute Carrier Family 14 Member 2) which showed highest expression change (down regulation) with fold change (log_2_) of ‘ <−2.0’ on at least two infection groups. In this study, we investigated all the above candidate genes and found their role in disease progression. We have listed these genes and their role in below headings.

ANPEP, called as Gene Aminopeptidase N (APN), is metallopeptidase that exerts strong influence on various immune response mechanisms. For example, APN has been known to cause decomposition of cytokines and peptides used by neurons^48–50^, and acts as receptor for viruses^51,52^. In addition, relation between expression level of APN and stimulated T-cell was reported^53^. Recently, it was suggested that APN controlled the balance of innate immune and adaptive immune by regulating TLR4 signal transduction pathway in myeloid cells^54^.

BGLAP, also known as Osteocalcin, is a noncollagenous protein, mainly found in bone, which needs vitamin K for its synthesis. This protein was thought to play a role in calcium ion homeostasis and used as biological marker for bone formation^55^. In addition, it concerns in endocrine regulation, especially in digestive system, by stimulating release of insulin hormone from β-cell of the pancreas and adiponectin hormone from fat cells, respectively^56^. As well as these function, it has been reported to take a role in promotion of energy availability and sexual maturation of male by stimulation of testosterone biosynthesis^57,58^.

CMC4, called as MTCP1, has been mainly reported to be related in various diseases. It was reported that MTCP1 gene affected T-cell homeostasis prior to process of leukemogenesis in transgenic mice^59^. Although the function of this gene has been entirely discovered, regulation error of MTCP1 gene affected on cell survival and cell growth^60^. Besides, this gene was known to be related in the pathogenesis of a subset of T-cell lymphoproliferative diseases^61,62^.

COL1A1 and COL1A2 encodes the pro-α1 chain and the pro-α2 chain protein, respectively. The type I collagen, which is comprised by two pro-α1 chains and one pro-α2 chain, plays a role in reinforcement and support in most of all connective tissues such as bone, cartilage, skin, and tendon, and offers those tissues rigidity and elasticity^63,64^. This protein was reported to stimulate expression of pro-inflammatory cytokines and professional phagocytes in teleost fish gilthead seabream^65,66^. In addition, it has been reported that receptor-mediated interaction which is formed in between cells and collagen molecule might affect in wound healing, inflammatory, and immune response by activating various factors such as cytokines, growth factor, and matrix metalloprotease^63,66–70^.

SLC14A2, also is known Urea transporter 2 (HUT2), is important gene involved in urea transport and play role in physiology. In mammals, two types of urea transporter (SLC14A1 and SLC14A2) has been reported^71^ and were regulated by vasopressin hormone^72^. The kidney uses urea to maintain the appropriate concentration and volume of blood. Without control of these proteins, organism would result in extreme damage in urinary system. Besides, a previous study has reported that genetic variation including nucleotide change is known to significantly influence blood pressure (BP) and metabolism syndrome^73,74^.

As shown in Supplementary Table 2, immune-related DEGs were revealed as results of three pathogens infection. Infection of pathogens caused activation of immune system to respond to invasion of harmful external elements, indicated that change of expression level of immune-related genes. The down-regulation of gene could sequentially influence on expression of various molecules positioned in down-streams in metabolic pathway. In this view, CD13, which is one of down-regulated genes by infection of three pathogens on common, was reported to inactivate interleukin 8^49^. Representative function of this cytokine is to induce migration of neutrophils and granulocytes toward infection site. In addition, absence of CD13 considerably improves cross-presentation of soluble antigen via regulation of receptor-mediated uptake^75^. Thus, decrease of CD13 expression consequentially might activate immune response in the olive flounder. MTCP1 gene induced malignant T-cell transformation^59^ and was related in the leukemogenic process of mature T-cell proliferation^61^. This gene was thought to maintain balance of immune response by T-cell. The innate immune system mediates the initial inflammatory response by pathogen infection or injury. For rapid response against external pathogens, infected cells secrete various cytokines to induce effector cells and complements. The type I collagen, which is comprised by proteins coded from COL1A1 and COL1A2, was involved in the expression of pro-inflammatory cytokines in the innate immune system. However, two genes (COL1A1 and COL1A2) showed decreasing expression pattern after infection in our study. Given sampling period (7 days from infection) of olive flounders for this study, it might be explained that the adaptive immune response was activated in the olive flounder genome. It is hard to understand comprehensively about immune system of fish genome. However, our results were expected to contribute for further study by extend of genomic knowledge in the olive flounder.

In conclusion, this study is helpful in understanding infection of the diversified pathogens (antigenic variation) and their role in disease progression in the olive flounder. The differentially expressed genes identified from transcriptome analysis using three types of pathogens could be useful to study the basic diagnosis and therapeutic mechanisms, and offer opportunities for designing the appropriate vaccines or drug targets for pathogen specific candidate genes. Because of lack of genomic information or using one external infection factor, previous studies have been limited to understand global expression pattern of whole genes in the olive flounder genome. We hope that this research would contribute to achieve great outcome in various biological field.

## Materials and Methods

### Ethical statement

All experiments with the olive flounders in this study were carried out in accordance with the guidelines and regulation approved by Ethical Committee of Pukyong National University.

### Preparation of olive flounder gill tissues

Gill tissues from twelve olive flounder (BW = ~50 g, n = 3/group) including healthy and infected fish with each pathogen were used for this study. Briefly, healthy fish (non-challenged), sampled fish at 7 days post challenge (dpc) with *S. parauberis* at 5.06 × 10^3^ CFU/fish in 1/3 seawater of 21 °C, sampled fish at 7 dpc with VHSV at 106 PFU/fish in 1/3 seawater of 19 °C, and sampled fish at 26 days post exposure to *M. avidus* at 3 × 10^3^ cells/ml in 1/3 seawater of 20 °C, respectively, were used in this study. We used total twelve samples including three samples per each group (uninfected, virus-infected, bacteria-infected, and parasite-infected) to minimize difference of genomic feature shown from individuals and understand common pattern of gene expression change in each group.

### RNA preparation for NGS

The samples were stored in RNAlater RNA Stabilization (Qiagen, Germany) solution. RNeasy Mini Kit columns were used to extract total RNA according to the manufacturer’s protocol (Qiagen). In order to measure RNA quality, an Agilent 2100 Bioanalyzer using the RNA 6000 Nano Chip (Agilent Technologies, Amstelveen, The Netherlands) was used. Integrity values to RNA measurement were confirmed by the RNA integrity number (RIN). Total RNA quantity was assessed with Infinite 200 PRO NanoQuant Spectrophotometer (TECAN, Switzerland).

### Construction of cDNA libraries for transcriptome analysis

Building of transcriptome libraries were conducted by Illumina’s TruSeq RNA protocol, and 1–2 μg of total RNA were used in each samples. AMPure XP beads (BECKMAN COULTER) and Ambion Fragmentation Reagents kit (Ambion, Austin, TX) were used for extraction of Poly(A)+ RNA and their fragment, respectively. As the following steps, cDNA synthesis, end-repair, A-base addition, and ligation of the Illumina indexed adapters were carried out according to Illumina’s protocol. The size-selected 250–300 bp cDNA fragments were loaded on a 3% Nusieve 3:1 (Lonza) agarose gel for libraries. The cDNA fragments were recovered using QIAEX II gel extraction reagents (Qiagen), and amplified using Phusion DNA polymerase (New England Biolabs) for 14 PCR cycles. The amplified libraries were purified by AMPure XP beads, their concentration and product sizes were assessed on an Agilent 2100 Bioanalyzer. Sequencing of paired-end libraries were conducted with the Illumina HiSeq2500, (2 × 100 nucleotide read length).

### Transcriptome analysis and differential gene expression

Transcriptome analysis were carried out with the RNAseq Tuxedo protocol. Mapping of Sequences were conducted against the Olive flounder draft genome (Submitted at present) using TopHat v2.0.9 with default options for paired-end sequences. Transcripts expression were estimated using the Cufflinks program v2.1.1. Total sequencing reads were subjected to preprocessing as follows: adapter trimming was performed using cutadapt with default parameters, and quality trimming (Q30) was performed using FastQC with default parameters. Processed reads were mapped to the Olive flounder draft genome (Submitted at present) using tophat and cufflink with default parameters^76^. The differential analysis was performed using Cuffdiff^76^ using default parameters. Further, the FPKM values from Cuffdiff were normalized and quantitated using R Package Tag Count Comparison (TCC)^77^ to determine statistical significance (e.g., P values) and differential expression (e.g., fold changes.). Through these statistics analysis, we sorted DEGs having *p* < 0.05 and showed them as results.

### Gene ontology analysis

DEG set for GO analysis was acquired from transcriptome analysis. DEGs were annotated from InterProScan database and non-redundant protein database in the NCBI. DAVID and uniprot tool were used for exploring the functional enrichment of these DEGs and sorting specially out the immune-related GO terms with *p*-value of <0.05, respectively.

## Electronic supplementary material

Supplementary table 1

Supplementary table 2

Supplementary table 3

Supplementary Figure 1
