# Supplementary material for: Gene expression profiles alteration after infection of virus, bacteria, and parasite in the Olive flounder (Paralichthys olivaceus)
Source: Sci Rep. 2018 Dec 24;8:18065. doi: 10.1038/s41598-018-36342-y (PMC6305387; doi:10.1038/s41598-018-36342-y)
Supplement: Supplementary file 4 — Supplementary Figure 1 [file 41598_2018_36342_MOESM4_ESM.doc]

**Gene expression profiles alteration after infection of virus, bacteria, and parasite**

**in the Olive flounder (*Paralichthys olivaceus*)**

Gyu-Hwi Nam^1,2^, Anshuman Mishra^2^, Jeong-An Gim^3^, Hee-Eun Lee^1,2^, Ara Jo^1,2^, Dahye Yoon^4^, Ahran Kim^5^, Woo-Jin Kim^6^, Kung Ahn^7^, Do-Hyung Kim^5^, Suhkmann Kim^4^, Hee-Jae Cha^8^, Yung Hyun Choi^9^, Chan-Il Park^10^, and Heui-Soo Kim^1,2,*^


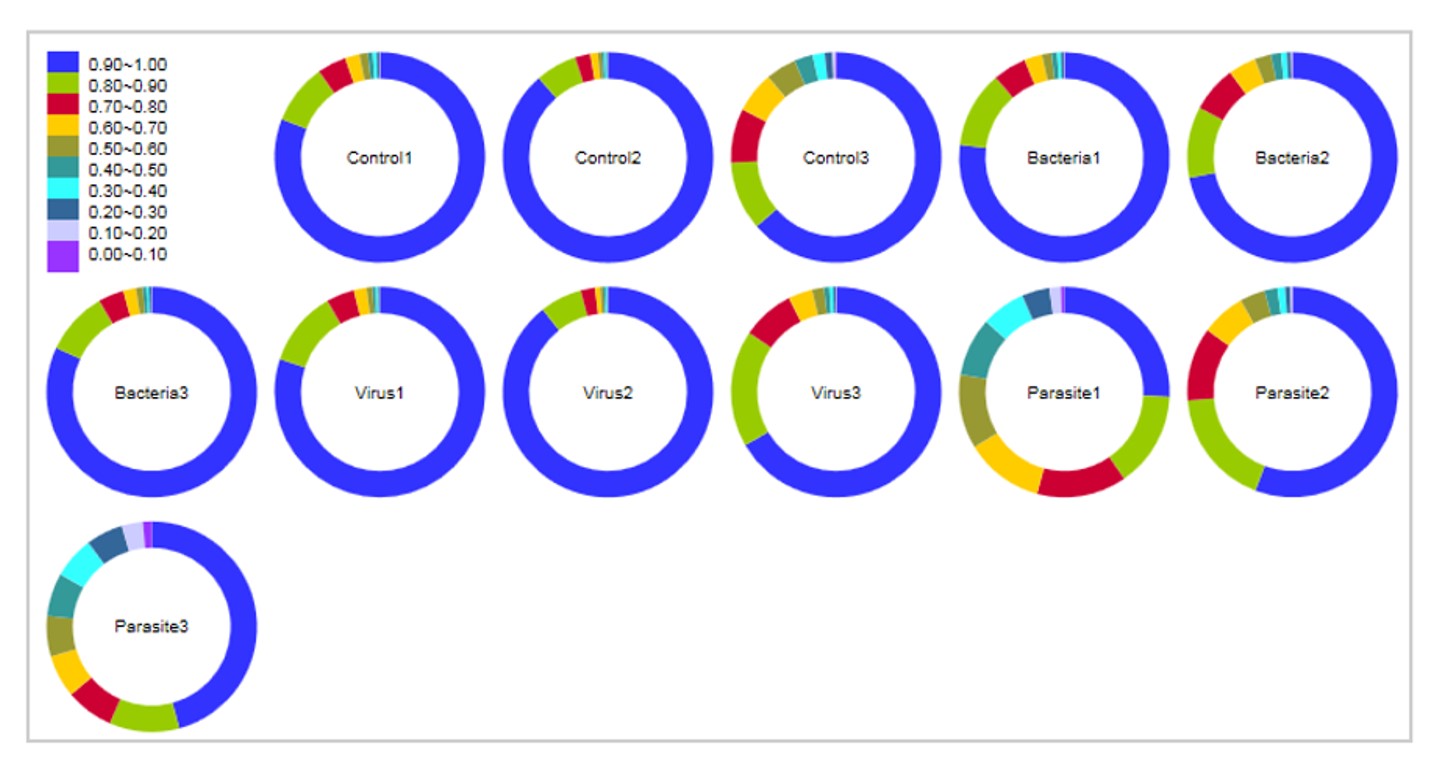


**Supplementary figure 1. Gene coverage of the reads from twelve infection samples mapped to the draft genome.** Results are shown as the ratio among the ranks per sample.
